# Host EPAC1 Modulates Rickettsial Adhesion to Vascular Endothelial Cells via Regulation of ANXA2 Y23 Phosphorylation

**DOI:** 10.3390/pathogens10101307

**Published:** 2021-10-12

**Authors:** Zhengchen Su, Thomas R. Shelite, Yuan Qiu, Qing Chang, Maki Wakamiya, Jiani Bei, Xi He, Changcheng Zhou, Yakun Liu, Emmanuel Nyong, Yuejin Liang, Angelo Gaitas, Tais B. Saito, Bin Gong

**Affiliations:** 1 Department of Pathology, University of Texas Medical Branch, Galveston, TX 77555, USA; zhengchensu@gmail.com (Z.S.); yuqiu@utmb.edu (Y.Q.); qichang@utmb.edu (Q.C.); jibei@utmb.edu (J.B.); drhexi@163.com (X.H.); zhoucc_uro@163.com (C.Z.); yakun1210@hotmail.com (Y.L.); ecnyong@utmb.edu (E.N.); 2 Division of Infectious Disease, Department of Internal Medicine, University of Texas Medical Branch, Galveston, TX 77555, USA; trshelit@utmb.edu; 3 Department of Neurology, University of Texas Medical Branch, Galveston, TX 77555, USA; mawakami@utmb.edu; 4 Department of Microbiology and Immunology, University of Texas Medical Branch, Galveston, TX 77555, USA; yu2liang@utmb.edu; 5 The Estelle and Daniel Maggin Department of Neurology, Icahn School of Medicine at Mount Sinai, 1468 Madison Ave, New York, NY 10029, USA; angelo.gaitas@mssm.edu; 6 The Vector-Pathogen-Host Interaction Unit, Laboratory of Bacteriology, Rocky Mountain Laboratories, National Institute of Allergy and Infectious Diseases, National Institutes of Health, Hamilton, MT 59840, USA; 7 Sealy Center for Vector Borne and Zoonotic Diseases, University of Texas Medical Branch, Galveston, TX 77555, USA; 8 The Center of Biodefense and Emerging Infectious Diseases, University of Texas Medical Branch, Galveston, TX 77555, USA

**Keywords:** bacterial adhesion, rickettsia, endothelial cell, *EPAC1*, annexin A2, fluidic force microscopy, single living cell

## Abstract

Introduction: Intracellular cAMP receptor exchange proteins directly activated by cAMP 1 (*EPAC1*) regulate obligate intracellular parasitic bacterium rickettsial adherence to and invasion into vascular endothelial cells (ECs). However, underlying precise mechanism(s) remain unclear. The aim of the study is to dissect the functional role of the *EPAC1-ANXA2* signaling pathway during initial adhesion of rickettsiae to EC surfaces. Methods: In the present study, an established system that is anatomically based and quantifies bacterial adhesion to ECs in vivo was combined with novel fluidic force microscopy (FluidFM) to dissect the functional role of the *EPAC1-ANXA2* signaling pathway in rickettsiae–EC adhesion. Results: The deletion of the *EPAC1* gene impedes rickettsial binding to endothelium in vivo. Rickettsial OmpB shows a host *EPAC1*-dependent binding strength on the surface of a living brain microvascular EC (BMEC). Furthermore, ectopic expression of phosphodefective and phosphomimic mutants replacing tyrosine (Y) 23 of *ANXA2* in *ANXA2*-knock out BMECs results in different binding force to reOmpB in response to the activation of *EPAC1*. Conclusions: *EPAC1* modulates rickettsial adhesion, in association with Y23 phosphorylation of the binding receptor *ANXA2*. Underlying mechanism(s) should be further explored to delineate the accurate role of cAMP-*EPAC* system during rickettsial infection.

## 1. Introduction

Rickettsioses are devastating human infections [1]. These arthropod-borne diseases are caused by obligate intracellular bacteria of the genus *Rickettsia* (*R.*), including *R. rickettsii* [2,3] and *R. parkeri* [4,5,6] that cause Rocky Mountain spotted fever and *R. parkeri* rickettsiosis [7], respectively; *R. conorii*, the causative agent of Mediterranean spotted fever [8]; and *R. australis*, which is a part of the transitional group (TRG) [9] and causes Queensland tick typhus [10]. A licensed vaccine is not available. Microvascular endothelial cells (ECs) are the primary targets of infection, and edema resulting from EC barrier dysfunction occurs in brain and lung in most cases of lethal spotted fever group (SFG) and TRG rickettsial infections in humans [1,2,9]. Typically, rickettsial infections are controlled by appropriate broad-spectrum antibiotic therapy if diagnosed early [2,3]. However, rickettsial infections can cause nonspecific signs and symptoms rendering early clinical diagnosis difficult [11,12]. Untreated or misdiagnosed *R* infections are frequently associated with severe morbidity and mortality [1,3,13,14,15]. Although doxycycline is the antibiotic of choice for *R* infections, it only stops bacteria from reproducing, but does not kill the rickettsiae. A fatality rate as high as 32% has been reported in hospitalized patients with Mediterranean spotted fever [15]. It is forecasted that global climate change will lead to more widespread distribution of rickettsioses [16,17]. Comprehensive understanding of rickettsial pathogenesis is urgently needed for the development of novel therapeutics [6,14,18,19,20,21,22].

cAMP is one of the most common and universal second messengers. cAMP regulates a myriad of important biological processes under physiological and pathological conditions. Therefore, current pharmaceutical medications target the cAMP signaling pathway more than any other [23]. cAMP translates environmental signals into regulatory effects in cells [24,25,26,27,28]. As such, several microbial pathogens have evolved a set of diverse virulence-enhancing strategies that exploit the cAMP signaling pathways of their hosts [29,30,31,32,33,34]. cAMP-based cell signaling mediated by intracellular cAMP receptors, exchange proteins directly activated by cAMP (EPAC)1 and 2, is one of major contributors to the transduction of the effects of cAMP [35,36,37]. In ECs, *EPAC1*, not *EPAC2*, is the dominant isoform [38,39,40]. We have been interested in host cAMP signaling pathways for their possible roles in rickettsial infection and reported that both genetic depletion and pharmacological inactivation of the host *EPAC1* protected mice from fatal rickettsioses [29]. Our ex vivo and in vitro evidence further support that the inhibition of intracellular *EPAC1* suppressed rickettsial adherence to and invasion into ECs [29]. However, underlying precise mechanism(s) remain to be dissected.

The mechanism(s) underlying bacterial adherence to vascular endothelial cells (ECs) under shear stress from flowing blood is critical to our understanding the initial stages of the pathogenesis of endovascular bacterial infections. The constitutively expressed native proteins on and/or in the mammalian host plasma membrane play crucial functional role(s) during the initial stage of infection, just prior to EC activation. Recently we reported that endothelial surface annexin A2 (*ANXA2*), a well-characterized plasminogen and plasminogen activator receptor [41,42], functions as a receptor for SFG [9] and TRG [43] rickettsial adhesin outer membrane protein B (OmpB) binding and is also involved in establishing *Staphylococcus aureus* adhesion to EC surfaces [44]. Meanwhile, we reported that intracellular *EPAC1* modulates *ANXA2* tyrosine (Y) 23 phosphorylation, and inactivation of *EPAC1* suppresses *ANXA2* expression on the EC luminal surface by downregulating Y23 phosphorylation [45]. We are now extending these works to determine whether the identified *EPAC1-ANXA2* signaling pathway is involved in the regulation of rickettsial adhesion to ECs.

In the present study, an established, anatomically based, in vivo quantitative system that measures bacterial adhesion to ECs [44] was combined with novel fluidic force microscopy (FluidFM) to dissect the functional role of the *EPAC1-ANXA2* signaling pathway during initial adhesion of rickettsiae to EC surfaces. We corroborate that the deletion of the *EPAC1* gene impedes rickettsial binding to endothelium in vivo. A study coupling FluidFM and site-directed mutagenesis with a single, living brain microvascular EC (BMEC) provides evidence to support that *EPAC1* governs rickettsial adhesion to EC surfaces in association with regulation of *ANXA2* Y23 phosphorylation.

## 2. Results

### 2.1. In Vivo Corroborating Rickettsial Adhesion to EC Surfaces in an EPAC1-Dependent Manner

**Figure 1:**
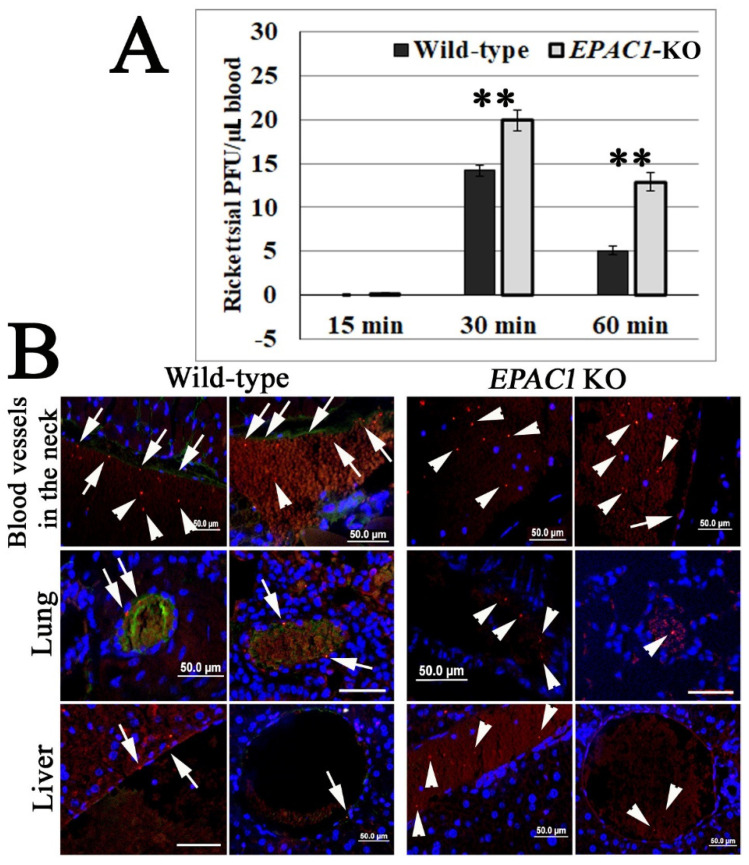
Global depletion of *EPAC1* blocks rickettsial adherence to blood vessel luminal surfaces in vivo. (**A**) Plaque assay for *R. australis* using blood samples collected from the orbital venous sinus of wild-type (WT) (n = 10) and *EPAC1*-knock out (KO) (n = 11) mice at different times p.i. with 10 LD_50_ doses (1 × 10^7^ PFU) of *R. australis* given by the intravenous route. Data are represented as mean ± SEM. The data were analyzed using One- Way ANOVA. **: compared to the control group, *p* < 0.01. (**B**) Representative IF-based identification of rickettsiae (red) in WT (n = 5) and *EPAC1*-KO (n = 5) mice 60 min p.i. with 10 LD_50_ doses of *R. australis* given by the intravenous route. *EPAC1* is stained with green signals. Rickettsiae adhere to the intima layer of blood vessels (arrows) or wrap in blood clots (arrowheads) in the lumens of blood vessels. Nuclei are stained blue. Scale bars, 50 µm.

Our previous work provided in vitro and ex vivo evidence that *EPAC1* plays a critical regulatory role in the early stage of rickettsial invasion into nonphagocytic host cells [29]. Using our recently established in vivo quantitative system to measure bacterial adhesion to vascular ECs [44] in our *EPAC1*-knock out (KO) mouse model [45], we can now verify the regulatory role of *EPAC1* during rickettsial adhesion. Plaque assays reveal that the number of viable rickettsiae in circulating blood was higher in *EPAC1*-KO mice (n = 11) than wild-type (WT) mice (n = 10) at 30 min and 1 hr post-infection (p.i.) when the bacteria were given by the intravenous route (Figure 1A). Similar as our previous report [44], immunofluorescence staining showed that, in WT mice, rickettsiae were mainly detected on the luminal surface or tunica intima of the vascular wall (Figure 1B). However, in *EPAC1*-KO mice, fewer rickettsiae were detected in the same areas. In contrast, unattached rickettsiae were visible in blood clots in the lumen of these blood vessels (Figure 1B).

These observations suggest that rickettsial adhesion to the vascular luminal surface occurs in a host *EPAC1*-dependent manner.

### 2.2. Rickettsial OmpB Shows a Host EPAC1-Dependent Binding Strength on the Surface of a Living EC

**Figure 2:**
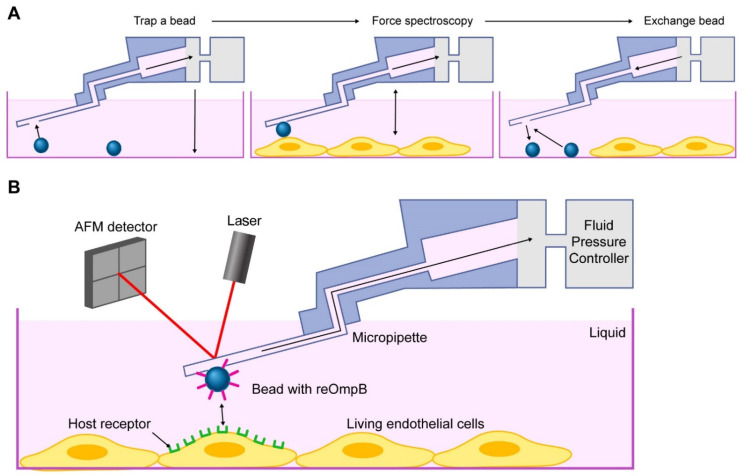
Fluidic force microscopy (FluidFM). Contamination and degradation of colloid surfaces limits the lifetime of a colloid probe and induces potential bias when the probe is reused to measure the same biomolecule in a different sample. A simple replacement of the colloid probe after experiments or contamination is impossible for conventional AFM. FluidFM was developed to conquer these limitations. (**A**) Operation moods of FluidFM system using micropipette-based cantilever, providing strong connections between the colloid and cantilever to use exchangeable colloid probes with the same cantilever. (**B**) Schematic representation of the quantification of irreversible interacting forces between the protein-coated colloid probe and the target living cell by the FluidFM system, mainly composed of fluid pressure controller and AFM detector.

Employing our in vivo model and atomic force microscopy (AFM), we found that rickettsiae mediate adhesion to the surface of ECs via host *ANXA2* [44]. Compared to traditional AFM using a protein-functionalized colloid cantilever to evaluate the protein–protein interaction, the micropipette cantilever of FluidFM enables us to use exchangeable colloid probes with the same cantilever for the quantification of irreversible interacting forces, drastically increasing the experiment throughput. Moreover, it reduces the bias from excessive usage of a single functionalized colloid (Figure 2) [46].

**Figure 3:**
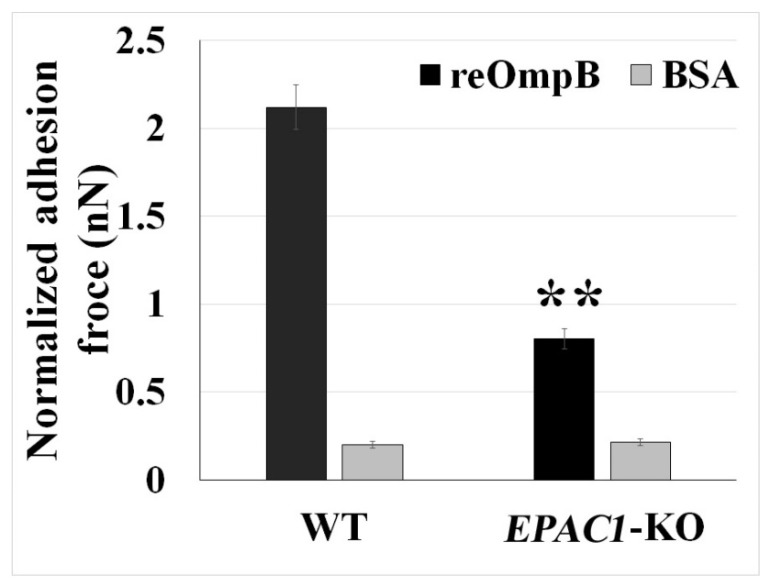
Rickettsial OmpB shows a host *EPAC1*-dependent binding strength on the surface of a living EC. FluidFM studies measure the binding forces (nanoNewton, nN) between reOmpB-coated microbead and a single living WT or *EPAC1*-KO mouse BMEC. Data are represented as mean ± SEM. The data were analyzed using One-Way ANOVA. **: compared to the group of WT, *p* < 0.01. At least three different detection areas were measured in one cell. Ten cells were sampled per group.

OmpB is a rickettsial adhesin responsible for host invasion [47,48]. Using recombinant OmpB (reOmpB)-functionalized single microbead, we employed the force spectroscopy capacity of the FluidFM system to determine the binding strength between reOmpB and the surface of single living BMEC (Figure 3). A BSA-coated microbead was used as negative control [49]. As expected, reOmpB-coated single microbead generally exhibit higher adhesion forces to WT BMECs (*p* < 0.01). To examine whether *EPAC1* contributes to reOmp and BMEC binding, we compared the adhesion forces generated in WT versus *EPAC1*-KO BMECs; reduced adhesion forces between a reOmpB and a *EPAC1*-KO BMEC were recorded (*p* < 0.01) (Figure 3). Furthermore, we utilized mouse BMECs from WT and *EPAC1*-KO mice to compare endothelial surface-associated rickettsiae, which were examined by impermeable immunofluorescence (IF) microscopy [44]. All ECs were fixed with 4% paraformaldehyde as impermeable fixation for the impermeable IF microscopy. Compared to WT, cell surface-associated bacteria were decreased in *EPAC1*-KO BMECs at 15 min p.i. with 10 MOI rickettsiae (Figure S1).

**Figure 4:**
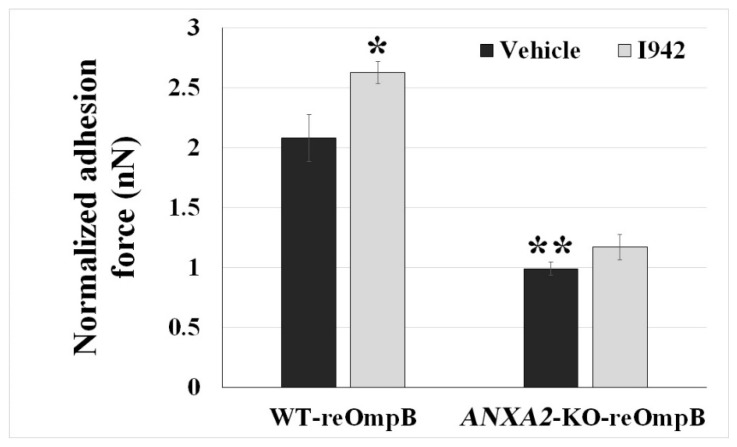
*EPAC1* specific activator enhances reOmpB binding force to single living EC dependent of *ANXA2.* FluidFM studies measured the binding forces (nanoNewton, nN) between reOmpB-coated microbead and single living WT or *ANXA2*-KO mouse BMEC, which was exposed to I942 (5 µM) or vehicle (DMSO at same amount as the I942 preparation in the media) for 24 h. Stock solution of I942 (5 mM) was added into culture media to a final concentration at 5 µM. Data are represented as mean ± SEM. The data were analyzed using One-Way ANOVA. *: compared to the group of WT-vehicle, *p* < 0.05. **: compared to the group of WT-vehicle, *p* < 0.01. At least three different detection areas were measured in one cell. Ten cells were sampled per group.

Taking advantage of a recently developed non-cAMP analogue, an *EPAC1* specific activator named I942 that competitively binds the cyclic nucleotide-binding domain of *EPAC1* [50,51,52], we observed that I942 at 5 µM in culture media enhanced the interacting force between reOmpB and a live WT BMEC (*p* < 0.05) (Figure 4). Depletion of *ANXA2* in ECs diminished reOmpB binding forces to the *ANXA2*-KO BMEC surface (*p* < 0.01) (Figure 4), corroborating our previous report that anti-*ANXA2* antibody weakens the interacting force between reOmpB and a living EC [44]. Interestingly, depletion of *ANXA2* in ECs reduced the enhanced binding force between reOmpB and ECs in the presence of I942 (versus the WT group, *p* = 0.09), independent of its concentration in the culture medium (Figure S2).

Collectively, these data suggest that the *EPAC1-ANXA2* pathway is involved in the interaction of OmpB with ECs during the initial stages of bacterial binding.

### 2.3. Ectopic Expression of Phosphodefective and Phosphomimic Mutants Replacing Y23 of ANXA2 in ANXA2-KO BMECs Results in the Display of Different Binding Force to reOmpB in Response to the Activation of EPAC1

Our previous study suggested that *EPAC1* regulates endothelial luminal surface ANXA2 expression by modulating the phosphorylation of Y23 [45]. To identify whether Y23 is an *EPAC1*-targeted site in the N-terminus of *ANXA2* that leads to regulation of rickettsial adhesion, we transfected *ANXA2*-KO BMECs with mouse WT *ANXA2* construct, phosphodefective *ANXA2* mutant Y23F, or phosphomimic *ANXA2* mutant Y23E, respectively, which were generated by site-directed mutagenesis using their respective mutant oligonucleotides [53]. Expressions of *ANXA2* in these constructs-transfected *ANXA2*-KO BMECs were detected, respectively (Figure S3).

**Figure 5:**
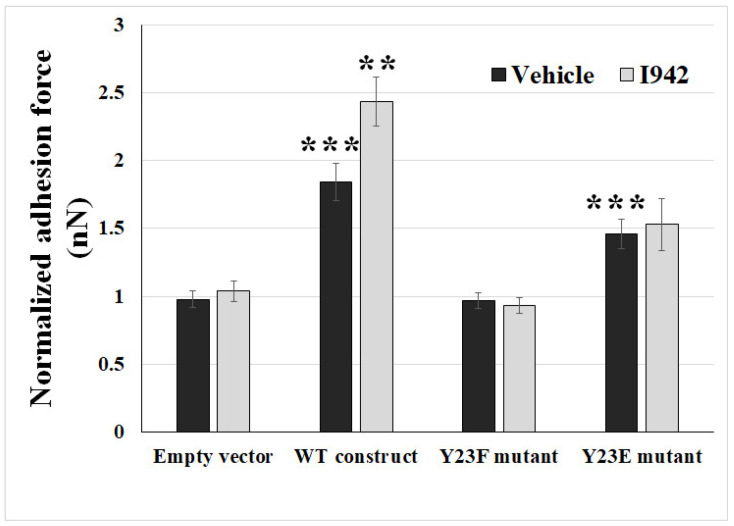
Host *EPAC1* governs rickettsial adhesion to EC surface via regulation on phosphorylation of Y23 in *ANXA2***.** FluidFM studies measured the binding forces (nanoNewton, nN) between reOmpB-coated microbead and single living *ANXA2*-KO mouse BMEC, which was transfected with WT *ANXA2* construct, phosphodefective *ANXA2* mutant Y23F, or phosphomimic *ANXA2* mutant Y23E, after exposure to I942 (5 µM) or vehicle for 24 h. The cells were transfected with empty vectors were used as negative controls. Data are represented as mean ± SEM. The data were analyzed using One-Way ANOVA. **: compared to the group of WT construct-vehicle, *p* < 0.01; ***: compared to the group of Empty vector-vehicle, *p* < 0.001. At least three different detection areas were measured in one cell. Ten cells were sampled per group.

Ectopic expression of the WT construct (versus no construct group, *p* = 5.36 × 10^−8^) and phosphomimic Y23E (versus no construct group, *p* = 6.70 × 10^−5^) in *ANXA2*-KO BMECs rescued the diminished reOmpB and cell binding force, respectively, whereas the phosphodefective mutant Y23F (*p* = 0.90) failed to do so (Figure 5). Furthermore, ectopic expression of the mouse WT *ANXA2* construct also rescued *EPAC1*-specific activator I942-induced enhancement of the binding force in *ANXA2*-KO BMECs, while no such effect occurred in the empty vector controls.

We reported that depletion of *ANXA2* reduces endothelial surface-associated rickettsiae [44]. In the present study we also utilized mouse BMECs from *ANXA2*-KO mice to compare the quantities of endothelial surface-associated rickettsiae. The amount of *ANXA2*-KO cell surface-associated bacteria were recovered at different levels after transfections with WT constructs or mutant Y23 constructs (Figure S1).

Collectively, these data suggest that host *EPAC1* governs rickettsial adhesion to EC surfaces via regulation of phosphorylation of Y23 in *ANXA2*.

## 3. Discussion

As an obligate intracellular bacterium, rickettsiae need to initiate adherence to host cells prior to infection. OmpB is the most abundant membranous protein in SFG and TRG rickettsia and functions as an adhesin during attachment and invasion [43,54]. Recently, we reported that *EPAC1* plays a critical role during rickettsial invasion [29] and identified endothelial *ANXA2* as a novel receptor for rickettsial OmpB binding to [44]. In the present study, we corroborated rickettsial adhesion to EC surfaces in an *ANXA2*- and *EPAC1*-dependent manner in vivo. Our FluidFM assay, employing a single exchangeable colloid-based micropipette, provided further evidence of host *EPAC1*-dependent binding of rickettsial OmpB on the surface of a living EC. In addition, our single living cell study that coupled FluidFM and site-directed mutagenesis showed that Y23 in the N-terminus of *ANXA2* is an *EPAC1*-targeted site involved in modulating rickettsial adhesion.

*ANXA2* in the cellular membrane compartment is believed to be regulated mainly via phosphorylation/dephosphorylation of the N-terminal domain of *ANXA2*, including S11, Y23, and S25 [55,56]. Phosphorylation of Y23 was identified as a regulatory switch during association of *ANXA2* with lipid rafts [45,57] and translocation of *ANXA2* between different subcellular compartments [45,58]. After phosphorylation of Y23 via the Src family tyrosine kinase(s)-dependent pathway, *ANXA2* is translocated to the cell surface in the format of heterotetramer with S100A10 by a yet unknown mechanism [57,58,59]. We reported that inactivation of *EPAC1* down-regulates Y23 phosphorylation of *ANXA2*, associated with *ANXA2* endothelial surface translocation, and does not impact the constitutive level of von Willebrand factor (vWF) in the plasma or other major hematological parameters [45]. Specifically, another *ANXA2* phosphodefective mutant Y23A, not *ANXA2* phosphomimic mutant Y23E, yields a predominantly negative effect for the translocation of *ANXA2* to the membrane surface. The Y23A mutant can still bind to S100A10 but only in the cytosol, not in the plasma membrane [60]. Given that *ANXA2* functions as a binding receptor for rickettsial OmpB, in the present study we further reveal that *EPAC1* regulates rickettsial adhesion to EC surfaces by targeting the *ANXA2* Y23. Depletion of *ANXA2* in ECs reduced the enhanced binding force between reOmpB and ECs in the presence of I942. The WT *ANXA2* construct rescued *EPAC1*-specific activator I942-induced enhancement of reOmpB binding forces to a *ANXA2*-KO BMEC surface. Furthermore, Y23E mutant rescued the diminished reOmpB and cell binding force in *ANXA2*-KO BMEC, whereas the Y23F mutant failed. However, there was no increasing in the binding force in the phosphomimic Y23E mutant-transfected *ANXA2*-KO BMEC in response to I942, further suggesting that *EPAC1* modulates Y23 phosphorylation of the binding receptor *ANXA2*. Currently the accurate pathway how *EPAC1* modulates the phosphorylation of specific residues(s) in *ANXA2* remains to be identified. Furthermore, the potential crosstalk between cAMP-*EPAC1* system and other identified factors involved in rickettsial adhesion and/or invasion should be investigated in future studies.

The colloidal probe-based AFM assay was developed to study protein–protein interactions and to quantify the interacting forces in the range from picoNewtons to nanoNewtons [46]. During manufacture, a colloid probe of different sizes is attached to a tipless cantilever by gluing and coated with recombinant proteins or antibodies by the user [44,45]. Entire colloidal probe-based cantilevers must be exchanged between experiments to measure different biomolecular interactions on the same sample with different protein functionalization. Contamination and degradation of colloid surfaces limits the lifetime of a colloid probe and induces potential bias when the probe is reused to measure the same biomolecule in a different sample. FluidFM was developed to conquer these limitations. A microfluidic pressure controller applies negative pressure to a connected hollow cantilever to quickly grab a functionalized microbead, and the microbead can be released by applying positive pressure through the micropipette channel [46]. Thus, FluidFM offers the following advantages: (1) it allows for the use of single exchangeable colloid for the quantification of irreversible and long-term protein-protein interactions, (2) it provides strong connections between the colloid and cantilever, (3) rapid exchange between microbeads with different protein coatings is possible, and (4) the method consistently uses the same reflex side of the same cantilever between different colloidal probes [46]. In the present study we use a reOmpB-coated microbead-based FluidFM cantilever to quantify the interaction between reOmpB and single, living cell in multiple experimental groups.

The binding forces between reOmpB and *EPAC1*-KO BMEC is still higher than the forces between BSA and *EPAC1*-KO BMEC. These data may suggest that intracellular *EPAC1* is not the sole regulator on endothelial surface receptor for OmpB-mediated rickettsial binding. Such unknown host surface protein(s) remain to be identified. Furthermore, although BSA has been widely employed as a mock control using AFM or FluidFM to measure protein-protein interaction in studying bacterial adhesion [49], identification of another recombinant Omp protein that exerts no binding force to a living BMEC will benefit establishing a sufficiently relevant negative control during FluidFM assay.

In conclusion, we have revealed a novel mechanism by which *EPAC1* modulates rickettsial adhesion, involving regulation of tyrosine 23 phosphorylation of the binding receptor *ANXA2*. This finding provides experimental and theoretical support for therapeutic strategies for rickettsiosis targeting the host cAMP-EPAC system. 

## 4. Materials and Methods

### 4.1. Mice

All animal experiments were performed according to protocols approved by the Institutional Animal Care and Use Committee of the University of Texas Medical Branch (UTMB). Wild-type (WT) mice (C57BL/6J) were obtained from The Jackson Laboratory (Bar Harbor, ME, USA). C57BL/6 *EPAC1*-KO mice were derived as described previously [45]. *ANXA2*-KO mice on the C57BL/6J background were a generous gift from Dr. Katherine Hajjar (Weill Cornell Medicine, New York, NY, USA) [61]. All mice used in this study were 8- to 12-week-old males. C57BL/6J mice are highly susceptible to *R. australis.* Therefore, this organism was chosen as the TRG rickettsial agent of choice [10].

### 4.2. Rickettsia and Cell Culture

*R. australis* (strain Cutlack) was prepared as described [29]. Uninfected Vero cells were processed as mock control material using the same procedure. All biosafety level (BSL-) 3 or ABSL-3 experiments were performed in CDC-certified facilities in the Galveston National Laboratory at UTMB (Galveston, TX, USA), using established procedures and precautions which were approved by the Institutional Biosafety Committee at UTMB.

Mouse brain microvascular endothelial cells (BMECs) were isolated from wild-type, *EPAC1*-KO, and *ANXA2*-KO mice using established protocol [44,51,62].

### 4.3. Antibodies and Other Reagents

A rabbit polyclonal antibody against SFG/TRG rickettsiae was obtained from the laboratory of Dr. David Walker (UTMB, Galveston, TX, USA). AlexaFluor 594-conjugated goat anti-rabbit IgG and ProLong Gold Antifade Reagent with DAPI were purchased from Invitrogen (Carlsbad, CA, USA). Recombinant rickettsial OmpB (reOmpB) (amino acids 1363–1655) was purchased from MyBioSource (San Diego, CA, USA). Unless otherwise indicated, all reagents were purchased from Thermo Fisher Scientific. Expression constructs for GFP-tagged mouse WT *ANXA2*, *ANXA2* mutant Y23E, and *ANXA2* mutant Y23F were purchased from GENEWIZ (South Plainfield, NJ, USA).

### 4.4. Cell Transfection

Transfections were performed using a published protocol [55]. Cells were transfected with of WT *ANXA2*, *ANXA2* Y23E, and *ANXA2* Y23F constructs (1 µg/500 µL for 1 × 10^5^ cells), respectively, using Magnetofection PolyMag and PolyMag Neo (OZ Biosciences, San Diego, CA, USA). The efficacy of transfection was evaluated with GFP fluorescent microscopy [55].

### 4.5. WT Construct Sequence (1020 bps)

5’-ATGTCTACTGTCCACGAAATCCTGTGCAAGCTCAGCCTGGAGGGTGATCATTCTACACCCCCAAGTGCCTACGGGTCAGTCAAACCCTACACCAACTTCGATGCTGAGAGGGATGCTCTGAACATTGAGACAGCAGTCAAGACCAAAGGAGTGGATGAGGTCACCATTGTCAACATCCTGACAAACCGCAGCAATGTGCAGAGGCAGGACATTGCCTTCGCCTATCAGAGAAGGACCAAAAAGGAGCTCCCGTCAGCGCTGAAGTCAGCCTTATCTGGCCACCTGGAGACGGTGATTTTGGGCCTATTGAAGACACCTGCCCAGTATGATGCTTCGGAACTAAAAGCTTCCATGAAGGGCCTGGGGACTGACGAGGACTCCCTCATTGAGATCATCTGCTCACGAACCAACCAGGAGCTGCAAGAGATCAACAGAGTGTACAAGGAAATGTACAAGACTGATCTGGAGAAGGACATCATCTCTGACACATCTGGAGACTTCCGAAAGCTGATGGTCGCCCTTGCAAAGGGCAGACGAGCAGAGGATGGCTCAGTTATTGACTACGAGCTGATTGACCAGGATGCCCGGGAGCTCTATGATGCCGGGGTGAAGAGGAAAGGAACCGACGTCCCCAAGTGGATCAGCATCATGACTGAGCGCAGTGTGTGCCACCTCCAGAAAGTGTTCGAAAGGTACAAGAGCTACAGCCCTTATGACATGCTGGAGAGCATCAAGAAAGAGGTCAAAGGGGACCTGGAGAACGCCTTCCTGAACCTGGTCCAGTGCATCCAGAACAAGCCCCTGTACTTCGCTGACCGGCTGTACGACTCCATGAAGGGCAAGGGGACTCGAGACAAGGTCCTGATTAGAATCATGGTCTCTCGCAGTGAAGTGGACATGCTGAAAATCAGATCTGAATTCAAGAGGAAATATGGCAAGTCCCTGTACTACTACATCCAGCAAGACACCAAGGGTGACTACCAGAAGGCACTGCTGTACCTGTGTGGTGGGGATGACTGA-3’.

For *ANXA2* mutant Y23E, the sequence of bases encoding Y23 is replaced with GAA. For *ANXA2* mutant Y23F, the sequence of bases encoding Y23 is replaced with UUC.

### 4.6. Mouse In Vivo, Anatomically-Based, Quantitative Rickettsial Adhesion Measurement System

The quantitative mouse in vivo rickettsial adhesion assay was performed as described [44]. The approach consists of two assays, the plaque assay to quantitatively measure viable bacteria in circulating blood, and the immunofluorescence staining (IF) assay to visualize the location of bacteria in the blood vessels 60 min p.i. The principal concept of the quantitative part is that the more rickettsiae adhere to the luminal surfaces of the blood vessels, the fewer rickettsiae are detected in peripheral blood samples. Multiple visceral organs are fixed without perfusion rinse (to keep blood in the vessel lumens) for IF-based histological studies to identify unattached rickettsiae, which are trapped in clots in vessel lumens [44]. The rule for the IF-based histological assay is to exclude capillaries during assessment because borders between blood and ECs were invisible in some capillaries after fixation. Briefly, after an ordinarily lethal dose of *R. australis* (1 × 10^7^ PFU/0.2 mL; the LD_50_ is 1 × 10^6^ PFU) was injected through the tail vein, rickettsial virulence (by plaque assay) was measured in a 1 µL blood sample collected from the orbital venous sinus (OVS) at different times until 1 h post-infection (p.i.). Rickettsial antigens and mouse *EPAC1* were detected with a rabbit polyclonal antibody against SFG/TRG rickettsiae (1:1000) and mouse monoclonal antibody against *EPAC1* (1:500) (clone 5D3, Cell Signaling Technology, Danvers, MA, USA), respectively, overnight at 4 °C, followed by incubation with AlexaFluor 594 goat anti-rabbit (1:1000) and AlexaFluor 488 goat anti-mouse (1:2000) antibodies for 15 min. Normal mouse or rabbit IgG was used as an antibody negative control during IF staining (Figure S4). Nuclei were counter-stained with DAPI. Fluorescent images were analyzed using an Olympus BX51 epifluorescence or Olympus IX81 confocal microscope.

### 4.7. FluidFM Adhesion Assay

Carboxylate modified latex microbeads (Invitrogen, Waltham, MA, USA) were coated with reOmpB using published protocols [63,64]. Briefly, microbeads were suspended in 2-(N-morpholino) ethanesulfonic acid (MES) buffer. Freshly made, water-soluble 1-ethyl-3-(3-dimethylaminopropyl) carbodiimide (EDAC) in MES (50 mg/mL) was added to the beads and incubated for 30 minutes. The beads were then washed twice in MES buffer with centrifugation. EDAC-activated beads were incubated with purified reOmpB at 50 µg/mL in phosphate-buffered saline (PBS) with gentle mixing at room temperature for 4 h. The vol/vol ratio between the beads and coating solution was 1/9 [63]. The bead-protein mixture was centrifuged and the supernatant was removed. The beads were then washed with PBS three times to completely remove free protein. Then, 0.4% glycine in 0.1M PBS was added to the beads for 10 min to deactivate the crosslinking, followed by one washing in 0.1M PBS. The beads were then resuspended in 0.1% glycine storage buffer. The coating procedure was performed under sterile conditions. Negative control BSA coated beads were created using the same procedure, but replacing reOmpB with BSA. 

The FluidFM system coupling Nanosurf Core AFM (NanoSurf, Liestal, Switzerland) and Fluidic Pressure Controller (Cytosurge AG, Glattbrugg, Switzerland) was used for this assay. A micropipette (2 µm, 0.3 N/m) (Cytosurge AG) was coated with 0.1 mg/ml poly(L-lysine)-*g*-poly(ethylene glycol) (PLL-g-PEG) to block nonspecific adhesions prior to calibration using the method of Sander et al. [65] and pre-set functions of the Nanosurf software. The air in the reservoir on the backside of the micropipette microchannel was removed by PBS prior to being connected to the Pneumatic Connector (Cytosurge AG). After subsequent connection to the Fluidic Pressure Controller, positive pressure (20 mBar) was applied to enable PBS to flow through the microchannel within the micropipette. The micropipette was then functionalized with an reOmpB-coated latex microbead by applying negative fluid pressure (−800 mBar) to trap the bead on the aperture of the micropipette. To measure the adhesion force between the layer of proteins that was coated on the surface of the bead and the surface of the single living cell, the bead-loaded micropipette was driven to approach the cell monolayer with 2 nN as the setpoint force, and paused on the surface of the cell for 0.5 min. This defined time established the interaction on the surface of the living cell [46]. The force spectroscopy was performed in the designated area (10 × 10 µm) to measure the unbinding force during rupture of the interaction between the ligands expressed in the designated areas at the apical surface of the single living cell and the reOmpB-coated microbead [45]. Ten cells were sampled per group. The bead was replaced following measurements of three cells to avoid nonspecific contamination.

### 4.8. Statistical Analysis

Values are reported as mean ± SEM. The data were analyzed using the Student’s *t*-test or One-Way ANOVA (Sigmaplot, Sigma Stat, Jandel Scientific Software, San Rafael, CA, USA). *p* values are as follows: ** *p* < 0.01 and * *p* < 0.05. Statistical significance is considered as *p* < 0.05.

## Data Availability

No new data were created or analyzed in this study. Data sharing is not applicable to this article.

## Supplementary Materials

The following are available online at https://www.mdpi.com/article/10.3390/pathogens10101307/s1, Figure S1: (A) Representative dual-target IF staining of rickettsiae (R.) (red) and EPAC1 (green) in mouse WT, EPAC1-KO, and ANXA2-KO BMECs 15 min post-infection (p.i.) using R. australis at a MOI of 10. Nuclei of BMECs were counterstained with DAPI (blue). The signals of EPAC1 are visualized (arrow heads) mainly in the nuclei. Scale bars: 100 µm. (B) Extracellular adhesive bacteria in WT and EPAC1-KO BMECs were enumerated by IF microscopy at 15 minutes p.i. using R. australis at a MOI of 10. Samples were blinded to the researcher doing the counting to avoid bias. All ECs were fixed with 4% paraformaldehyde as impermeable fixation for IF microscopy. n = 5 for all groups. **, compared to the WT group, p < 0.01. (C) Extracellular adhesive bacteria in WT and ANXA2-KO BMECs, which were transfected with different ANXA2 constructs, were enumerated by IF microscopy at 15 minutes p.i. using R. australis at a MOI of 10. All ECs were fixed with 4% paraformaldehyde as impermeable fixation for IF microscopy. n = 5 for all groups. Data are represented as mean ± SEM. *, compared to ANXA2-KO BMECs, p < 0.05, Figure S2: FluidFM studies measuring the binding forces (nanoNewton, nN) between a reOmpB-coated microbead and single living ANXA2-KO mouse BMEC, Figure S3: Expression of ANXA2 was examined using Western immunoblotting in mouse BMECs, Figure S4: Normal rabbit and mouse IgGs were used as reagent controls during the IF assay for tissues (left) and BMECs (right).
