# Supplementary material for: Host EPAC1 Modulates Rickettsial Adhesion to Vascular Endothelial Cells via Regulation of ANXA2 Y23 Phosphorylation
Source: Pathogens. 2021 Oct 12;10(10):1307. doi: 10.3390/pathogens10101307 (PMC8537355; doi:10.3390/pathogens10101307)
Supplement: Supplementary file 1 [file pathogens-10-01307-s001.zip › pathogens-1415085-supplementary.pdf]

SUPPLEMENTAL MATERIALS

Western immunoblotting

For western immunoblotting, equal amounts of soluble protein were subjected to 10% SDS–polyacrylamide gel electrophoresis (SDS-PAGE). Proteins were transferred onto a polyvinylidene difluoride membrane and then incubated with mouse anti-ANXA2 (1:5000) (clone 5, BD Bioscience, San Jose, CA) and anti-GAPDH (1:5000) (Thermo Fisher Scientific) at 4°C overnight, followed by incubation with a secondary antibody at 1:10,000 for 2 hrs. A goat anti-mouse or rabbit IgG and IgM (H+L)-HRP (Thermo Fisher Scientific) were used as the secondary antibodies. Blots were visualized using Pierce™ ECL Western Blotting Substrate kit (Thermo Fisher Scientific).

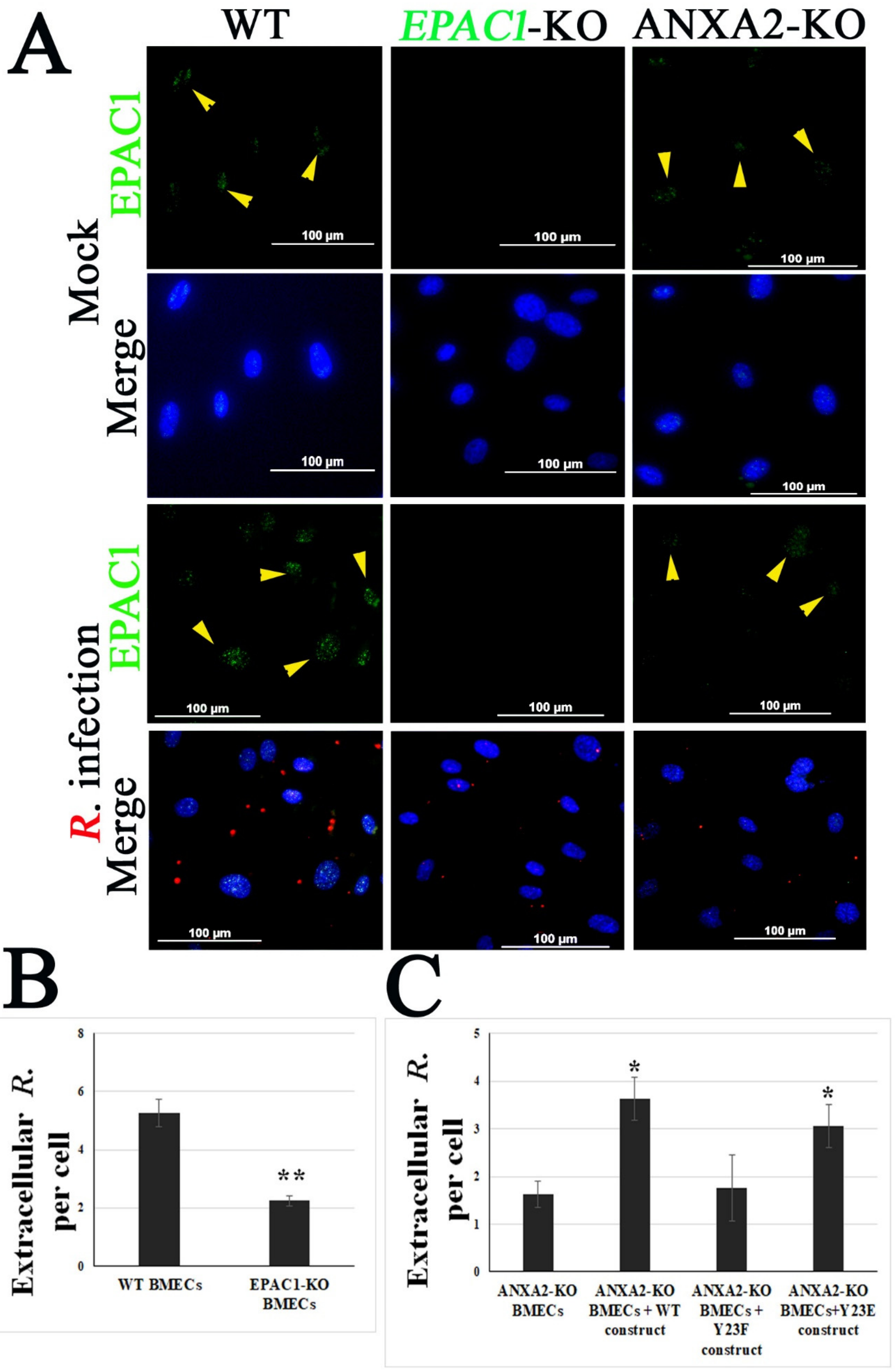

A
WT
EPAC1-KO
ANXA2-KO
Mock
EPAC1
Merge
R. infection
100 µm
B
Extracellular R. per cell
WT BMECs
EPAC1-KO BMECs
**
C
ANXA2-KO BMECs
ANXA2-KO BMECs + WT construct
ANXA2-KO BMECs + Y23F construct
ANXA2-KO BMECs+Y23E construct
*

Figure S1:(**A**) Representative dual-target IF staining of rickettsiae (*R.*) (red) and EPAC1 (green) in mouse WT, *EPAC1*-KO, and *ANXA2*-KO BMECs 15 min post-infection (p.i.) using *R. australis* at a MOI of 10. Nuclei of BMECs were counterstained with DAPI (blue). The signals of EPAC1 are visualized (arrow heads) mainly in the nuclei. Scale bars: 100 µm. (**B)** Extracellular adhesive bacteria in WT and *EPAC1*-KO BMECs were enumerated by IF microscopy at 15 minutes p.i. using *R. australis* at a MOI of 10. Samples were blinded to the researcher doing the counting to avoid bias. All ECs were fixed with 4% paraformaldehyde as impermeable fixation for IF microscopy. n = 5 for all groups. **, compared to the WT group, $p < 0.01$. (**C**) Extracellular adhesive bacteria in WT and *ANXA2*-KO BMECs, which were transfected with different ANXA2 constructs, were enumerated by IF microscopy at 15 minutes p.i. using *R. australis* at a MOI of 10. All ECs were fixed with 4% paraformaldehyde as impermeable fixation for IF microscopy. n = 5 for all groups. Data are represented as mean ± SEM. *, compared to *ANXA2*-KO BMECs, $p < 0.05$.

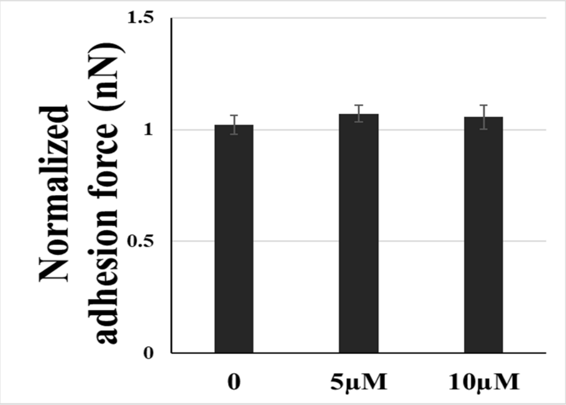

Figure S2: FluidFM studies measuring the binding forces (nanoNewton, nN) between a reOmpB-coated microbead and single living *ANXA2*-KO mouse BMEC, which was exposed to I942 at 0, 5, or 10 µM for 24 h. Data are represented as mean ± SEM. At least three different detection areas were measured in one cell. Ten cells per group were sampled.

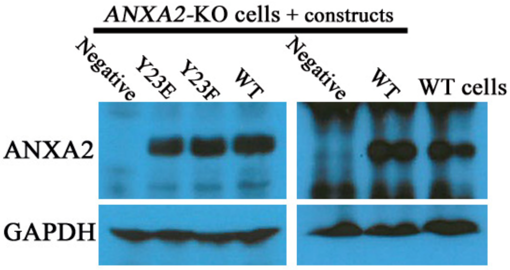

Figure S3: Expression of ANXA2 was examined using Western immunoblotting in mouse BMECs.

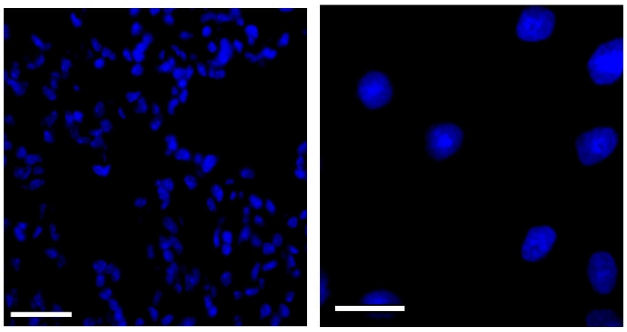

Figure S4: Normal rabbit and mouse IgGs were used as reagent controls during the IF assay for tissues (left) and BMECs (right). Nuclei were stained with DAPI. Scale bars: 50 μm.
